# The Effects of Inspiratory Muscle Training (IMT) on Patients Undergoing Coronary Artery Bypass Graft (CABG) Surgery: A Systematic Review and Meta-Analysis

**DOI:** 10.31083/j.rcm2401016

**Published:** 2023-01-09

**Authors:** Sisi Zhang, Bo Li, Xiaoping Meng, Houjuan Zuo, Dayi Hu

**Affiliations:** ^1^Division of Cardiology, Department of Internal Medicine, Tongji Hospital, Tongji Medical College, Huazhong University of Science and Technology, 430000 Wuhan, Hubei, China; ^2^Department of Thoracic Surgery, The Second Hospital of Jilin University, 130041 Changchun, Jilin, China; ^3^Department of Cardiovascular and Cardiac Rehabilitation, The Affiliated Hospital of Changchun Traditional Chinese Medicine, 130000 Changchun, Jilin, China

**Keywords:** inspiratory muscle training, coronary artery bypass graft surgery, pulmonary function, postoperative pulmonary complications, length of hospital stay

## Abstract

**Background:**

To determine the effects of inspiratory muscle training (IMT) alone on inspiratory muscle strength and endurance, pulmonary function, pulmonary complications, and length of hospital stay in patients undergoing coronary artery bypass graft surgery (CABG).

**Methods:**

We conducted a literature search across databases (Ovid MEDLINE(R) and Epub Ahead of Print, In-Process & Other Non-Indexed Citations and Daily; Ovid Embase; Ovid Cochrane Central Register of Controlled Trials; Ovid Cochrane Database of Systematic Reviews; and Scopus) from inception to December 2021. The eligibility criteria were randomized controlled trials that investigated the effects of IMT versus usual care or sham IMT in patients undergoing CABG.

**Results:**

A total of 12 randomized clinical trials with 918 patients were included in the meta-analysis. Postoperative IMT was associated with improved maximal inspiratory pressure (MIP), maximum inspiratory pressure (PImax), and six-minute walking test (6MWT) and with a decrease in length of hospital stay (LOS). For preoperative IMT, there was statistical significance between intervention and MIP, PImax, forced expiratory volume in one second (FEV1), forced vital capacity (FVC), postoperative pulmonary complications (PPCs), and LOS. Pre- and postoperative IMT resulted in improvements in MIP.

**Conclusions:**

Isolated IMT in patients who underwent CABG improved their inspiratory muscle strength and endurance, pulmonary function, and 6MWT and helped decrease postoperative pulmonary complications and the length of hospital stay.

## 1. Introduction

Coronary artery bypass graft (CABG) has become an option for treating coronary artery disease (CHD). Annually, more than 200,000 patients undergo CABG in the United States [1]. Despite the well-known benefits of CABG, many patients suffer from complications such as ventilatory muscle dysfunction, atelectasis, pneumonia, surgical site infection, and psychological disorders (anxiety, fear of death), which contribute to morbidity, mortality, and prolonged intensive care unit (ICU) stays [2, 3, 4]. Various forms of strategies such as aerobic training, endurance training, resistance training, and respiratory muscle training have demonstrated their benefits in inducing morphological and functional changes in the diaphragm while limiting the occurrence of postoperative pulmonary complications (PPCs) [5]. In addition, some devices such as positive expiratory pressure devices, and incentive spirometers are reported to prevent the incidence of PPCs [6]. Prehabilitation programs including nutrition support, smoking cessation, exercise interventions, and patient education have been suggested applied before surgery and may help reduce surgery-related complications, although no consensus has been reached.

The postoperative respiratory muscle weakness and phrenic nerve dysfunction that are common following CABG may contribute to pulmonary complications [7, 8]. Inspiratory muscle training (IMT) has been proposed as one form of respiratory physiotherapy; it uses progressive resistance loads provided by different devices to train with the aim of improving inspiratory muscle strength, endurance, and exercise capacity by activating the diaphragm [9, 10]. Therefore, IMT may help with recovery from CABG and reduce the incidence of postoperative pulmonary complications (PPCs).

To date, some systematic reviews have shown the potential beneficial effects of IMT for cardiac surgery. Cook *et al*. [11] assessed the effectiveness of IMT on postoperative hospital stay after CABG and/or heart valve surgeries. Kendall *et al*. [12] analyzed the effects of IMT, with the limited measured outcomes (PPCs, and length of hospital stay (LOS)) for patients undergoing upper abdominal or thoracic surgery, in addition to including pulmonary, and cardiac surgery. Recently, Dsouza *et al*. [13] investigated IMT in patients undergoing cardiac surgery, however, included limited articles and sample size. Furthermore, taking into account the different kinds of outcomes inherent to different types of surgery, it is possible that the effectiveness of IMT can be different. As the research has gradually shown increased risks of PPCs following CABG but existing evidence has not produced valuable conclusions, a comprehensive systematic review and meta-analysis of different phases of IMT as an isolated CABG intervention is deemed appropriate.

The purpose of this study was to review the effects of inspiratory muscle training as a stand-alone intervention during both the preoperative and postoperative periods on inspiratory muscle strength and endurance, pulmonary function, PPCs, and LOS in patients undergoing CABG.

## 2. Methods

This systematic review and meta-analysis were conducted under the Preferred Reporting Items for Systematic Reviews and Meta-Analyses (PRISMA) guidelines with the registration number CRD42020185136 [14].

### 2.1 Data Sources and Search Strategies

A comprehensive search was conducted of multiple databases from inception to December 2021 for studies limited to the English language and excluding animal studies. The databases included Ovid MEDLINE(R) and Epub Ahead of Print; In-Process & Other Non-Indexed Citations and Daily; Ovid Embase; Ovid Cochrane Central Register of Controlled Trials; Ovid Cochrane Database of Systematic Reviews; and Scopus.

The search strategy was designed and conducted by an experienced librarian with input from the study’s principal investigator. Controlled vocabulary supplemented with keywords was used to search for studies describing the effects of IMT in CABG. The actual strategy listing all search terms used and how they were combined is available in **Supplementary Table 1** online.

### 2.2 Eligibility Criteria 

Studies were included if they met the following criteria: randomized controlled trials published in English regardless of the publication date with populations of all ages and either sex with a history of CABG and receiving center-based, home-based, or community-based IMT. In the analyses, IMT before CABG, after CABG, and both before and after CABG was compared with usual care and sham IMT.

Nonhuman studies, nonrandomized controlled trials, abstracts, books, dissertations, study protocols, and case reports were excluded. Subjects who were scheduled for CABG or who only stated that they were undergoing cardiac surgery were excluded. IMT combined with other exercise interventions such as aerobic/resistance training or respiratory muscle strength training was also excluded.

The main outcomes of interest were pulmonary function, respiratory muscle strength and endurance, exercise capacity, PPCs, and LOS.

### 2.3 Selection Process

An experienced librarian searched for articles relevant to this review, and then two authors independently reviewed all titles and abstracts. The reference lists were also screened for other potentially eligible studies. These two authors assessed the full texts of all selected articles to verify if they met the inclusion criteria for the review. Disagreements between them were resolved by another senior author until the final decision was reached by consensus.

### 2.4 Data Extraction

Data extraction was undertaken using a standard form adapted from the Cochrane Collaboration by two authors [15]. The form included the basic characteristics of each study: publication date, country, sample size, average age, sex, intervention period (preoperative, postoperative, and preoperative and postoperative), outcomes, and intervention characteristics (device, intensity, training time, frequency and duration, supervision).

### 2.5 Quality Assessment 

The quality assessment of all eligible articles was conducted by two independent authors using the physiotherapy evidence database (PEDro) scale, which is a typical tool for evaluating the quality of physical therapy and rehabilitation studies [16]. The PEDro scale consists of 11 items based on a 9-item Delphi list [17]. The score range is 0 to 10, as 1 item on the PEDro scale (eligibility criteria) is related to external validity and is not used to calculate the score [18]. A PEDro score of 6–10 indicates high quality, a score of 4–5 indicates fair quality, and a score of <4 indicates poor quality [19].

### 2.6 Statistic Analysis

Review Manager 5.1 software (RevMan 2011, Cochrane Collaboration, Copenhagen, Denmark) was used for the data analysis [20]. Pooled-effect estimates were obtained by comparing the least square mean percentage change from baseline to study end for each group. The effect size was expressed as the mean difference (MD) with a 95% confidence interval (CI), due to the diversity of methodologies. An MD higher than 0.5 indicated a moderate effect size, while an MD higher than 0.8 indicated a large effect size [21]. We used either a fixed-effect model or a random-effect model depending on the heterogeneity. Four comparisons were made: combined exercise with IMT versus the exercise group, preoperative IMT versus the control group, postoperative IMT versus the control group, and both preoperative and postoperative IMT versus the control group. A *p*-value of ≤0.10 was regarded as significant. Cochran’s Q-test and the inconsistency statistic $I^{2}$ were used to assess heterogeneity. $I^{2}$ greater than 50% was considered indicative of high heterogeneity [22].

## 3. Results

### 3.1 Study Selection

**Fig. 1.**
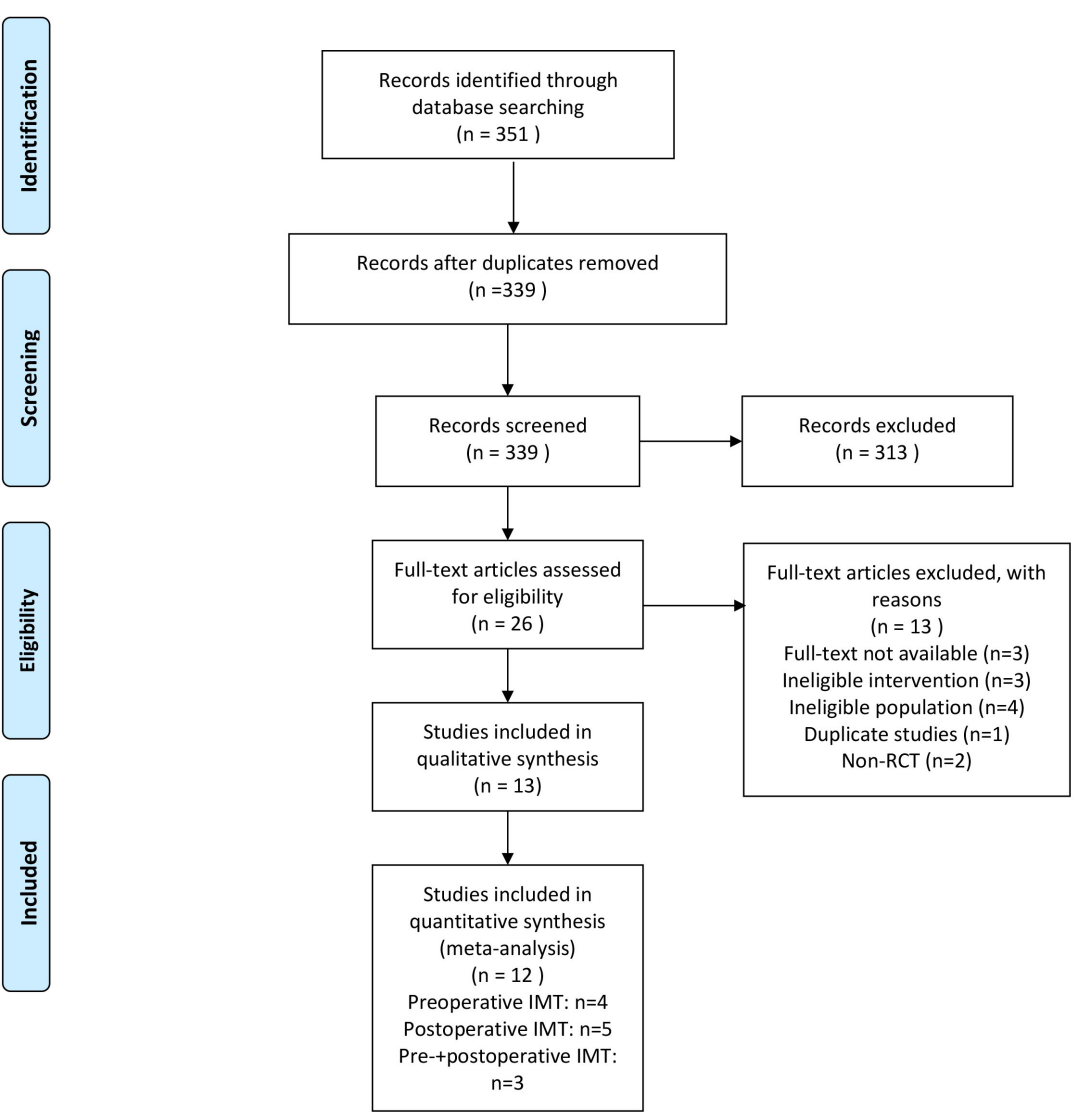
**PRISMA flow chart**.

We identified 351 articles from the database without additional resources. After the removal of duplicates, 339 articles were screened out. The remaining 26 full texts were retrieved and assessed for potential eligibility; of these, 12 RCTs were included in the meta-analysis [23, 24, 25, 26, 27, 28, 29, 30, 31, 32, 33, 34]. Four studies performed preoperative IMT [23, 24, 25, 34], five were in the postoperative period [26, 27, 28, 30, 31], and three compared preoperative and postoperative IMT with a control group [29, 32, 33]. The specific study selection flow chart is shown in Fig. 1.

### 3.2 Characteristics of the Included Studies

The included studies provided data on 918 patients, with sample sizes ranging from 20 to 279. According to the IMT period, 624 patients were allocated to preoperative IMT, 185 to postoperative IMT, and 109 to preoperative and postoperative IMT (Table 1).

**Table 1.** **Characteristics of the included studies**.

Trial	Country	Sample	Intervention Group	Control Group	Outcome	Ref
Weiner *et al*. 1998	Israel	IG: 42 (Female 26 Male 58; Age 59.2 ± 3.8 years)	Preoperative	Usual care	Primary outcomes: FVC, FEV1, PH, ${\mathrm{PaO}}_{2}$, ${\mathrm{PaCO}}_{2}$, MIP, Pmpeak/PImax	[23]
		CG: 42 (Age 63.8 ± 3.1 years)			Secondary outcomes: PPC	
Hulzebos *et al*. 2006	Netherlands	IG: 140 (Female 31 Male 108; Age 66.5 ± 9 years)	Preoperative	Usual care	Primary outcomes: MIP, Pmpeak/PImax, PPC	[24]
		CG: 139 (Female 30 Male 107; Age 67.3 ± 9.2 years)			Secondary outcomes: LOS	
Hulzebos *et al*. 2006	Netherlands	IG: 14 (Female 7, Male 7; Age 70.14 ± 9.86 years)	Preoperative	Usual care	Primary outcomes: Compliance, adverse events, satisfaction, motivation cardiovascular stress, heart rate, blood pressure, MIP	[25]
		CG: 12 (Female 6, Male 6; Age 70.5 ± 10.1 years			Secondary outcomes: PPC, LOS and lung function, FVC, FEV1, IVC	
Stein *et al*. 2009	Brazil	IG: 10 (Female 4 Male 6; Age 64 ± 7 years)	Postoperative	Usual care	Outcomes: FEV1, FVC, PImax, PEmax, PH, ${\mathrm{PaO}}_{2}$, ${\mathrm{PaCO}}_{2}$, heart rate, 6MWT, peak ${\mathrm{VO}}_{2}$, ${\mathrm{VE/VO}}_{2}$, ${\mathrm{VE/VCO}}_{2}$	[26]
		CG: 10 (Female 5 Male 5; Age 63 ± 6 years)				
Praveen *et al*. 2009	India	IG: 15 (Age 57.2 ± 5.62 years)	Postoperative	Usual care	MVV, PImax	[27]
		CG: 15 (Age 55.6 ± 5.26 years)				
Barros *et al*. 2010	Brazil	IG: 23 (Female 4 Male 19; Age 62.13 ± 8.1 years)	Postoperative	Usual care	Outcomes: MIP, MEP, PEF, TV, Dyspnea (Borg scale of dyspnea), Pain (VAS of pain), LOS	[28]
		CG: 15 (Female 9 Male 6; Age 67.08 ± 7.11 years)				
Savci *et al*. 2011	Turkey	IG: 22 (Female 3 Male 19; Age 62.82 ± 8.69 years)	Preoperative and Postoperative	Usual care	Outcomes: PPC, LOS, Ventilation time, MIP, MEP, 6MWT, Nottingham Health Profile, HADS	[29]
		CG: 21 (Female 2 Male 19; Age 57.48 ± 11.48 years)				
Matheus *et al*. 2012	Brazil	IG: 23 (Female 5 Male 18; Age 61.83 ± 13.53 years)	Postoperative	Usual care	Outcomes: MIP, MEP, TV, VC, PEF	[30]
		CG: 24 (Female 8 Male 16; Age 63.3 ± 10.2 years)				
Cordeiro *et al*. 2016	Brazil	IG: 25 (Female 14 Male 11; Age 56.4 ± 13 years)	Postoperative	Usual care	Outcomes: MIP, 6MWT, LOS	[31]
		CG: 25 (Female 9 Male 16; Age 57 ± 14.7 years)				
Elmarakby *et al*. 2016	Egypt	IG: 17 (Age 56.9 ± 3.75 years)	Preoperative and Postoperative	Usual Care	Outcomes: MIP, A-a gradient, ${\mathrm{SpO}}_{2}$.	[32]
		CG:16 (Age 56.95 ± 3.75 years)				
Turky *et al*. 2017	Egypt	IG: 17 (Age 56.9 ± 3.75 years)	Preoperative and Postoperative	Usual Care	Primary outcomes: ${\mathrm{PaO}}_{2}$, ${\mathrm{PaCO}}_{2}$, MIP, A-a gradient	[33]
		CG: 16 (Age 56.95 ± 4.35)			Secondary outcomes: ${\mathrm{SpO}}_{2}$.	
Valkenet *et al*. 2017	Netherlands	IG: 119 (Age 66 ± 9.2 years)	Preoperative	Usual Care	Outcomes: pneumonia, LOS, health-related QoL	[34]
		CG: 116 (Age: 67.5 ± 9.7 years)				

IG, Intervention group; CG, Control group; FEV1, forced expiratory volume in one second; FVC, forced vital capacity; MIP, maximal inspiratory pressure; MEP, maximal expiratory pressure; PH, hydrogen ion concentration; ${\mathrm{PaCO}}_{2}$, partial pressure of carbon dioxide, arterial; ${\mathrm{PaO}}_{2}$, partial pressure of oxygen, arterial; TV, tidal volume; VC, vital capacity; PEF, Peak Expiratory Flow; ${\mathrm{VE/VO}}_{2}$, ventilatory equivalents for oxygen; ${\mathrm{VE/VCO}}_{2}$, ventilatory equivalents for carbon dioxide; A-a gradient, alveolar-arterial oxygen gradient; MVV, Maximal Voluntary Ventilation; PImax, Maximum Inspiratory Pressure; PEmax, maximal expiratory pressure; LOS, length of hospital stay; PPC, postoperative pulmonary complication; QoL, quality of life; 6MWT, six-minute walking test; peak ${\mathrm{VO}}_{2}$, peak oxygen uptake; HADS, Hospital Anxiety and Depression Scale; ${\mathrm{SpO}}_{2}$, peripheral oxygen saturation.

### 3.3 Characteristics of the Interventions

Table 2 presents the characteristics of the interventions included. For the IMT, most of the studies used a threshold IMT device except for two: one of these used an expiratory positive airway pressure (EPAP) mask [26], and the other used a DHD respiratory trainer [27]. Patients started the training with a load from 15% to 40% of maximal inspiratory pressure (MIP). IMT frequency varied between studies from twice daily to 7 days per week. The duration of training also varied from 15 minutes to 30 minutes, and the total training length varied depending on whether IMT was administered in the preoperative or the postoperative period. It is common to provide supervision during the process of training.

**Table 2.** **Summary of intervention of IMT in included studies**.

Trial	Device	Intensity	Training time (minutes/day)	Frequency and duration	Ref
Weiner *et al*. 1998	Threshold Inspiratory Muscle Trainer, Health-scan, NJ, USA	15% of MIP	30	6 days/week, 2–4 weeks	[23]
Hulzebos *et al*. 2006	Inspiratory threshold-loading device (threshold IMT)	30% of MIP	20	7 days/week, 2–4 weeks	[24]
Hulzebos *et al*. 2006	Inspiratory threshold-loading device (threshold IMT)	30% of MIP	20	7 days/week, at least 2 weeks	[25]
Stein e*t al. *2009	Expiratory positive airway pressure mask	5–8 ${\mathrm{cmH}}_{2}$O	5–8	Once daily, 6 days	[26]
Praveen *et al*. 2009	DHD respiratory trainer	RPE >5	-	7 days/week, 15 days	[27]
Barros *et al*. 2010	Threshold inspiratory muscle trainer, Healthscan Products Inc.	40% of MIP	-	7 days/week, until discharge	[28]
Savci *et al*. 2011	Threshold Inspiratory Muscle Training, Respironics, Pittsburg, PA, USA	15% of MIP	30	7 days/week, 5 days	[29]
Matheus *et al*. 2012	IMT Respironics® Threshold®	40% of MIP	30	7 days/week, 3 days	[30]
Cordeiro *et al*. 2016	A pressure linear load device (Threshold® Resppironics®IMT)	40% of MIP	30	7 days/ week, 7–8 days	[31]
Elmarakby *et al*. 2016	Inspiratory threshold-loading device (ITLD, Powerbreathe Wellness Plus, Gaiam Ltd, Warwickshire, UK)	30% of MIP	15	Twice daily, until discharge	[32]
Turky *et al*. 2017	Inspiratory threshold-loading device (Powerbreathe Plus, Powerbreathe International, Warwickshire, UK)	30% of MIP	-	7 days/week, until discharge	[33]
Valkenet *et al*. 2017	Threshold IMT, Respironics New Jersey Inc., Cedar Grove, NJ, USA	30% of MIP	20	Once daily, until surgery	[34]

### 3.4 Quality Assessment of the Included Studies

We used the PEDro scale to assess the study methodologies, and the total scores varied from 4 to 8 points. Five studies were of high quality (PEDro score higher than 5) [24, 25, 26, 29, 32], while seven were of moderate quality (5 or 4) [23, 27, 28, 30, 31, 33, 34]. Most studies did not report a concealed allocation process (N = 9) [24, 27, 28, 30, 31, 32, 33, 34], and none of the included studies reported the blinding of subjects. The PEDro scores for the included studies are presented in Table 3.

**Table 3.** ** Quality assessment using the PEDro scale**.

Study	1*	2	3	4	5	6	7	8	9	10	11	Total
Cordeiro *et al*. 2016 [31]	Y	N	N	Y	N	N	N	N	Y	Y	Y	4
Valkenet *et al*. 2017 [34]	Y	Y	N	N	N	N	N	Y	Y	Y	Y	5
Matheus *et al*. 2012 [30]	Y	N	N	Y	N	N	N	Y	N	Y	Y	4
Barros *et al*. 2010 [28]	Y	Y	N	Y	N	N	N	N	N	Y	Y	4
Praveen *et al*. 2009 [27]	Y	Y	N	Y	N	N	N	N	N	Y	Y	4
Savci *et al*. 2011 [29]	Y	Y	Y	Y	N	N	N	Y	N	Y	Y	6
Stein *et al*. 2009 [26]	Y	Y	Y	Y	N	N	N	Y	Y	Y	Y	7
Hulzebos *et al*. 2006 [24]	Y	Y	N	Y	N	Y	Y	Y	Y	Y	Y	8
Elmarakby *et al*. 2016 [32]	Y	Y	N	Y	N	Y	N	Y	N	Y	Y	6
Turky *et al*. 2017 [33]	Y	Y	N	Y	N	N	N	Y	N	Y	Y	5
Hulzebos EHJ *et al*. 2006 [25]	Y	Y	Y	Y	N	N	Y	Y	Y	Y	Y	8
Weiner *et al*. 1998 [23]	N	Y	N	Y	N	N	N	N	N	Y	Y	4

1, eligibility criteria; 2, random allocation; 3, concealed allocation; 4, baseline comparability; 5, blind subjects; 6, blind therapists; 7, blind assessors; 8, adequate follow-up; 9, intention-to-treat analysis; 10, between-group comparisons;11, point estimates and variability. *Item 1 does not contribute to the total score.

### 3.5 Outcome Measures 

#### 3.5.1 Inspiratory Muscle Strength and Endurance

**Fig. 2.**
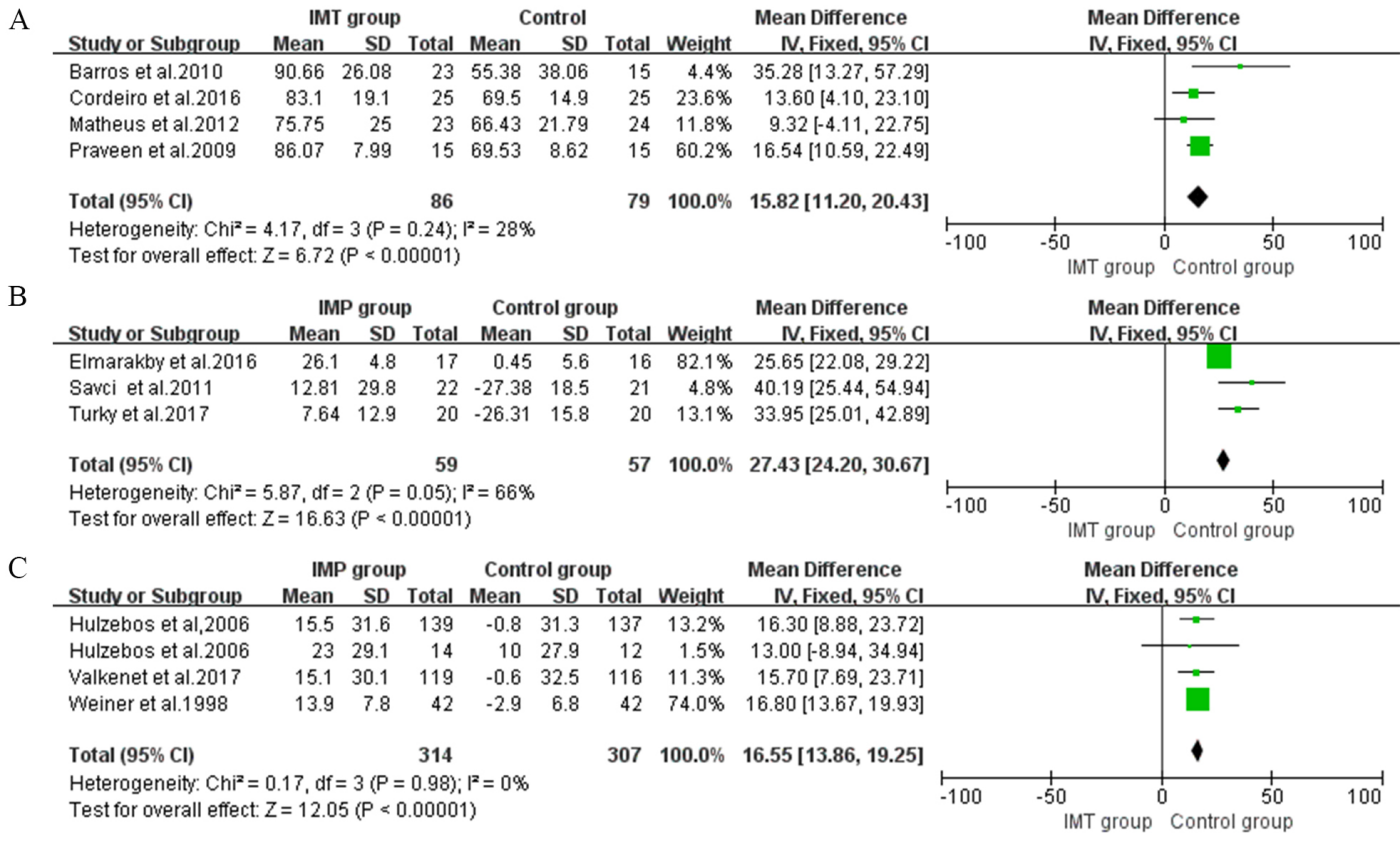
**Forrest plot of the effect of IMT on inspiratory muscle strength and endurance**. (A) The effect of postoperative IMT on maximal inspiratory pressure (MIP). (B) The effect of pre- and postoperative IMT on MIP. (C) The effect of preoperative IMT on MIP.

In postoperative IMT, four studies presented positive effects on maximal inspiratory pressure (MIP) (MD 15.82, 95% CI, 11.2 to 20.43, $I^{2}$ = 28%) (Fig. 2A) [27, 28, 30, 31].

**Fig. 3.**
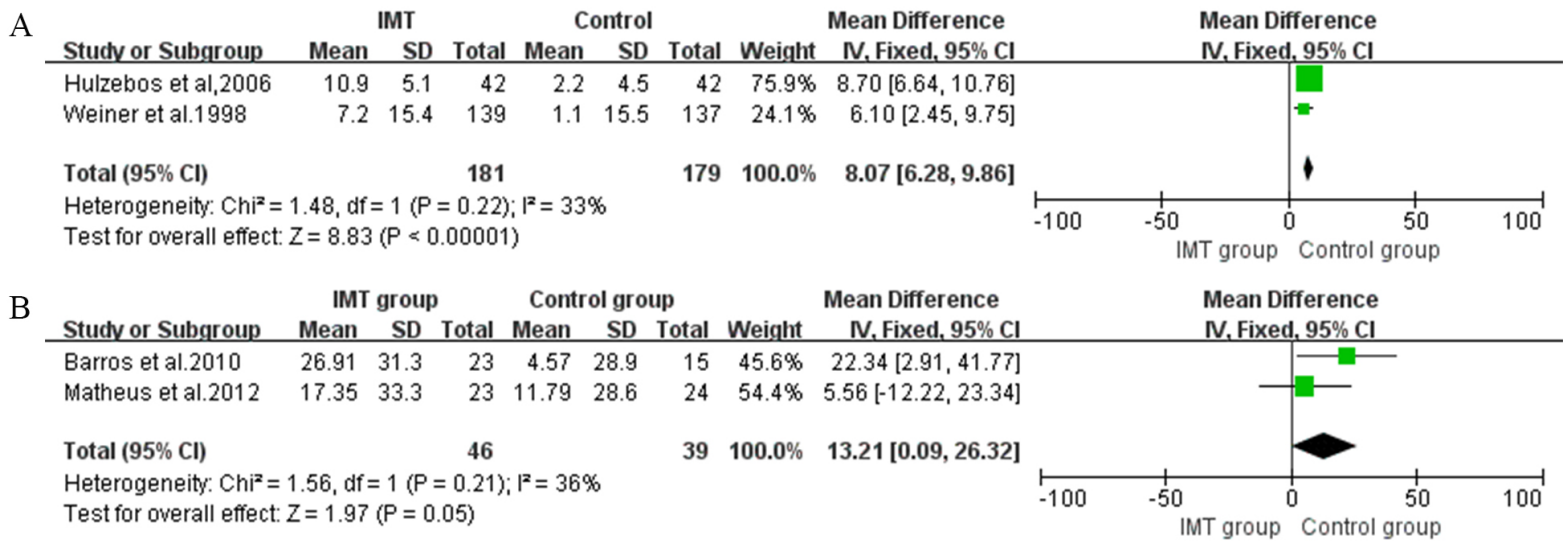
**Forrest plot of the effect of IMT on expiratory muscle strength**. (A) The effect of IMT on inspiratory muscle endurance/maximum inspiratory pressure (Pmpeak/PImax). (B) The effect of IMT on maximal inspiratory pressure (MEP).

The effect of preoperative IMT on MIP was assessed in four studies and showed benefits (MD 16.55, 95% CI, 13.86 to 19.25, $I^{2}$ = 0%) (Fig. 2C) [23, 24, 25, 34]. Two studies assessed inspiratory muscle endurance with inspiratory muscle endurance/maximum inspiratory pressure (Pmpeak/PImax) and showed a significant improvement of 8.07% (95% CI, 6.28 to 9.86, $I^{2}$ = 33%) compared with usual care (Fig. 3A) [23, 24].

Three studies that compared preoperative and postoperative IMT with control groups evaluated MIP and obtained a significant increase of 27.43 ${\mathrm{cmH}}_{2}$O (95% CI, 24.2 to 30.67, $I^{2}$ = 66%) (Fig. 2B) [29, 32, 33].

#### 3.5.2 Expiratory Muscle Strength

The expiratory muscle strength is quantified by maximal inspiratory pressure (MEP). A total of two postoperative IMT intervention studies were included in the meta-analysis, with significant differences in this outcome (MD 13.21, 95% CI, 0.09 to 26.32, $I^{2}$ = 36%) (Fig. 3B) [28, 30].

#### 3.5.3 Pulmonary Function 

**Fig. 4.**
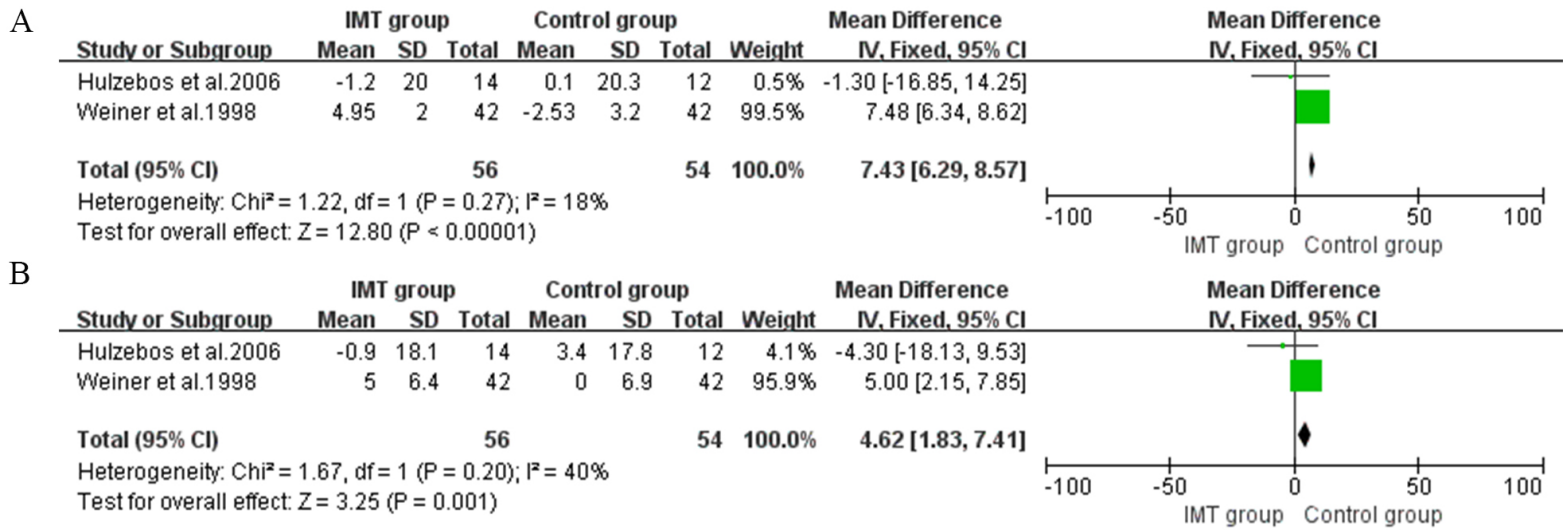
**Forrest plot of the effect of IMT on pulmonary function**. (A) The effect of IMT on forced expiratory volume in one second (FEV1). (B) The effect of IMT on forced vital capacity (FVC).

In preoperative IMT, two studies assessed FEV1 and FVC as outcomes [23, 25]. The meta-analysis showed a significant improvement in FEV1 of 7.43% predicted (95% CI, 6.29 to 8.57, $I^{2}$ = 18%) (Fig. 4A) and in FVC of 4.26% predicted (95% CI, 1.83 to 7.41, $I^{2}$ = 40%) (Fig. 4B).

#### 3.5.4 Postoperative Pulmonary Complications

**Fig. 5.**
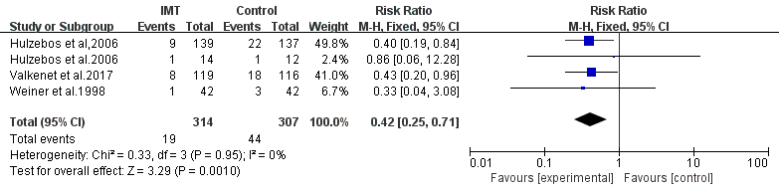
**Forrest plot of the effect of IMT on postoperative pulmonary complications**.

Four studies assessed PPCs as outcomes. Patients who received preoperative IMT had a reduced risk of PPCs (Risk ratio (RR) = 0.42, 95% CI, 0.25 to 0.71, $I^{2}$ = 0%) (Fig. 5) [23, 24, 25, 34].

#### 3.5.5 Duration of Hospital Stay 

**Fig. 6.**
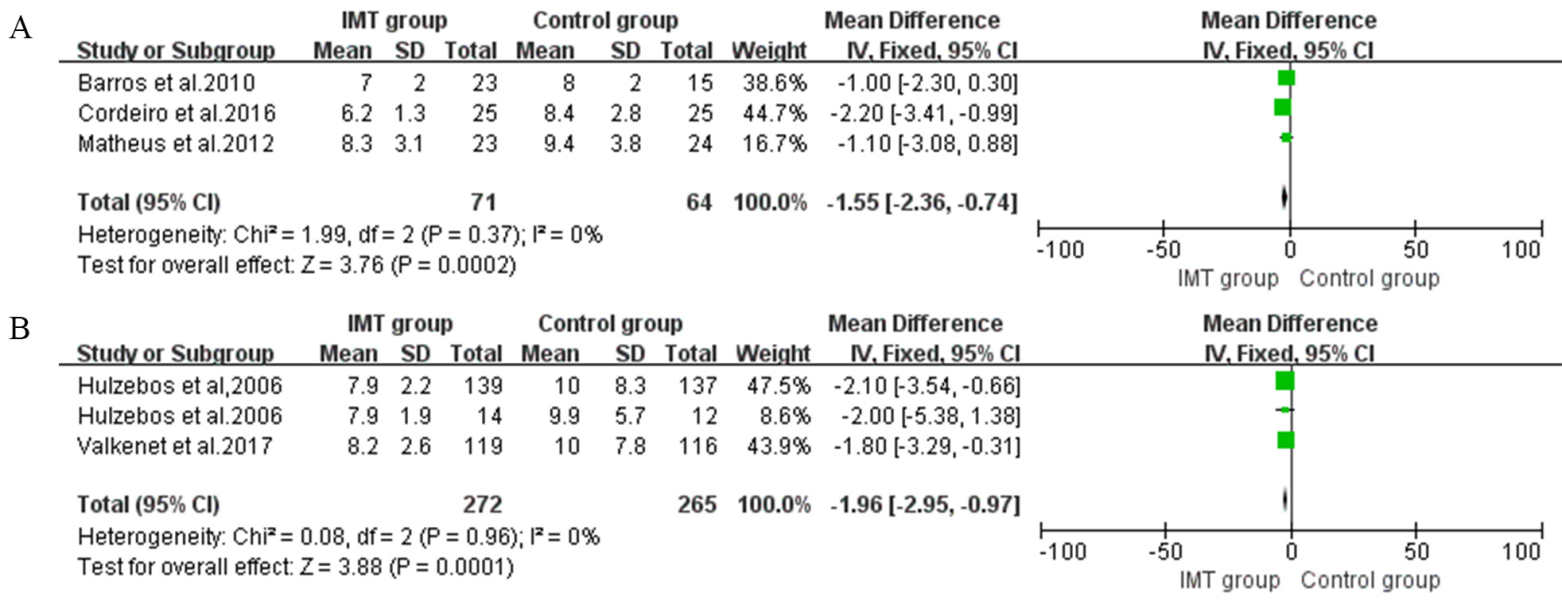
**Forrest plot of the effect of IMT on duration of hospital stay**. (A) The effect of preoperative IMT on length of hospital stay (LOS). (B) The effect of postoperative IMT on LOS.

Three studies in preoperative IMT and three studies in postoperative IMT presented data regarding the length of hospital stay [24, 25, 28, 30, 31, 34]. Overall, we observed a decrease in LOS due to preoperative IMT (MD –1.96, 95% CI, –2.95 to –0.97) (Fig. 6A) and a reduction of –1.55 days (95% CI, –2.36 to –0.74) for participants in postoperative IMT, with no heterogeneity ($I^{2}$ = 0) (Fig. 6B).

#### 3.5.6 Exercise Capacity

**Fig. 7.**
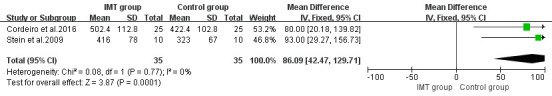
**Forrest plot of the effect of IMT on 6MWT**.

Only two studies reported data regarding exercise capacity using the 6MWT. The increase in 6MWT after postoperative IMT was significant compared with usual care (MD 86.09, 95% CI, 42.47 to 129.71, $I^{2}$ = 0%) (Fig. 7) [26, 31].

## 4. Discussion

The findings of this systematic review and meta-analysis are that in patients undergoing CABG, preoperative IMT improves inspiratory muscle strength, endurance, and pulmonary function and reduces PPCs and LOS. Postoperative IMT improves inspiratory muscle strength, endurance, and 6MWT and reduces LOS. Pre- and postoperative IMT benefits inspiratory muscle strength. This is the first systematic meta-analysis that included RCTs with isolated IMT in CABG populations rather than IMT performed in combination with other interventions. Previous systematic reviews did show evidence that pre- and postoperative IMT may improve inspiratory muscle strength and pulmonary function and reduce PPCs and LOS in patients undergoing cardiac surgery, which is in accordance with our findings [35].

### 4.1 Meta-Analysis of Inspiratory Muscle Strength and Endurance

For patients who underwent CABG, decreased inspiratory muscle strength represented as lower MIP and PImax was common and contributed to the incidence of PPCs after CABG [36, 37]. Respiratory muscles, similar to skeletal muscles, can be trained for strength and endurance [38, 39, 40]. In our findings regarding preoperative IMT, all included studies reported improved inspiratory strength or endurance expressed as Pmpeak/PImax. There were also improvements in MIP from pre- and postoperative IMT, although the heterogeneity was high ($I^{2}$ = 66%) due to the different protocols adapted in the intervention groups [29, 32, 33].

For postoperative IMT studies [26, 27, 28, 30, 31], benefits were shown for both MIP and MEP. Although IMT did not focus on expiratory muscles, postoperative training still affected MEP; the explanation for this was that strong inspiratory muscles were helpful in the elastic recoil of the lungs and chest wall by expanding the position of the thorax. Improved expiratory muscle strength benefits cough strength and effective airway clearance, which therefore delays the occurrence of PPCs [7].

### 4.2 Meta-Analysis of Pulmonary Function

CABG causes diaphragmatic dysfunction that leads to restrictive lung volumes, impaired ventilatory mechanics, and decreased lung compliance [12].

In our meta-analysis, only two studies investigated FEV1 and FVC, and both reported positive effects [23, 25]. After CABG, the pulmonary function decreased within two weeks. Previous studies demonstrated that pulmonary function decreased by 40% after CABG accompanied by a 25% decrease in ${\mathrm{PaO}}_{2}$ [41, 42]. The results in our study agree with the effects of IMT on pulmonary function in other populations: FVC and FEV1 are not directly affected by expiratory or inspiratory muscle strength but rather by lung size and expiratory airflow, which helps explain this study’s outcomes.

### 4.3 Meta-Analysis of Exercise Capacity

The analysis of exercise capacity included studies measuring 6MWT, and these showed benefits from postoperative IMT. IMT is established as an effective intervention that attenuates inspiratory muscle weakness and has been applied in the context of cardiovascular rehabilitation for heart failure [43]. Sinoway *et al*. [44] showed a minimum 6MWT increase of 25 m in patients with coronary artery disease who underwent IMT. Our study results show a 6MWT increase of 86.09 m, positive evidence that postoperative IMT intervention could improve exercise capacity [26, 31]. These results can be explained by the reduction in diaphragmatic overload associated with subsequently increased oxygen delivery to the limbs, which leads to increased exercise capacity [45, 46]. Inspiratory muscle weakness leads to a decrease in lung volume that contributes to exercise intolerance. Thus, improvements in inspiratory muscle strength and pulmonary function after IMT could also help increase exercise capacity.

### 4.4 Meta-Analysis of PPCs and LOS

It is known that prolonged hospital stays are associated with PPCs, morbidity, and mortality [47, 48]. These complications in turn increase the rate of hospital readmission, creating a vicious circle. Pooled data from the included studies show that inspiratory muscle training during the preoperative period reduced LOS (MD: 1.96 days) and PPCs (RR: 0.42) [23, 24, 25, 34]. The same effect was also observed during the postoperative period (MD: 1.55 days) [28, 30, 31]. For most of the related studies, IMT could effectively improve inspiratory muscle strength and pulmonary function, further helping to reduce the incidence of PPCs. Our findings are in accordance with previous meta-analysis findings showing that IMT can prevent PPCs such as atelectasis and pneumonia [12, 35].

Some outcomes help to explain how IMT reduces the risk of LOS and PPCs. The improvement in inspiratory muscle strength and pulmonary function can help with recovery from surgery, contributing to reducing the length of the hospital stay. In addition, forceful respiratory muscles assist with lung expansion and increase vital capacity and tidal volume, which, in turn, reduce the risk of pulmonary complications. The use of IMT for patients who undergo CABG can reduce the length of the hospital stay and pulmonary complications, in turn reducing the burden on the whole medical care system.

## 5. Limitations

There are several limitations to this meta-analysis. One, the IMT protocols included some variations in terms of duration, frequency, and a number of sessions. High homogeneity existed in this systematic review even though we adopted a random-effect model, and it was impractical to conduct sensitivity analysis or meta-regression due to the limited number of included studies. This was the main limitation of this review.

Additionally, however, due to the small sample size, the lack of patient and therapist blinding, and the diversity of the IMT protocols used in the selected studies, the evidence here must be carefully analyzed. Although we performed a rigorous search and screening process, the insufficient keywords and the language restriction limited the number of eligible studies. Despite the benefits of IMT, no clear statements can be made regarding an optimal protocol after CABG. Future studies are needed to identify the optimal IMT protocol in order to maximize its clinical benefits.

## 6. Conclusions

Isolated IMT interventions showed benefits in improving inspiratory muscle strength and endurance, pulmonary function, and 6MWT and reducing the pulmonary complications and length of hospital stay for patients undergoing CABG. Based on this evidence, it is worthwhile to use inspiratory muscle training as an intervention to prevent postoperative complications or to improve exercise capacity and quality of life within populations after CABG. More studies are needed to confirm these findings.

## Supplementary Material

Supplementary material associated with this article can be found, in the online version, at https://doi.org/10.31083/j.rcm2401016.
