# Supplementary material for: The Effects of Inspiratory Muscle Training (IMT) on Patients Undergoing Coronary Artery Bypass Graft (CABG) Surgery: A Systematic Review and Meta-Analysis
Source: Rev Cardiovasc Med. 2023 Jan 9;24(1):16. doi: 10.31083/j.rcm2401016 (PMC11270382; doi:10.31083/j.rcm2401016)
Supplement: Supplementary file 1 [file 2153-8174-24-1-016-s1.zip › Supplementary material.docx]

Supplementary Table 1. Actual Search Strategies

OVID

Database(s): **Ovid MEDLINE(R) 1946 to Present and Epub Ahead of Print, In-Process & Other Non-Indexed Citations and Ovid MEDLINE(R) Daily, EBM Reviews - Cochrane Central Register of Controlled Trials** March 2020**, EBM Reviews - Cochrane Database of Systematic Reviews** 2005 to December 1, 2021, **Embase** 1974 to 2020 Week 18

Search Strategy:

| **#** | **Searches** |
| --- | --- |
| 1 | breathing exercises/ or breathing exercise/ |
| 2 | ((exercis* or training) adj1 (breathing or respirat* or ventilatory)).mp. |
| 3 | "inspiratory muscle training".mp. |
| 4 | or/1-3 |
| 5 | exp heart surgery/ |
| 6 | cardiovascular surgical procedures/ |
| 7 | coronary artery bypass graft/ |
| 8 | coronary artery bypass/ |
| 9 | coronary artery disease/su |
| 10 | ((coronary or "aorta coronary" or "aorto coronary" or aortocoronary or heart or cardiac) adj1 (anastomosis or bypass or shunt* or graft* or surgery)).mp. |
| 11 | CABG.mp. |
| 12 | or/5-11 |
| 13 | 4 and 12 |
| 14 | limit 13 to english language [Limit not valid in CDSR; records were retained] |
| 15 | limit 13 to no language specified [Limit not valid in CDSR; records were retained] |
| 16 | 14 or 15 |
| 17 | limit 16 to (conference abstract or editorial or erratum or note or addresses or autobiography or bibliography or biography or blogs or comment or dictionary or directory or interactive tutorial or interview or lectures or legal cases or legislation or news or newspaper article or patient education handout or periodical index or portraits or published erratum or video-audio media or webcasts) [Limit not valid in Ovid MEDLINE(R),Ovid MEDLINE(R) Daily Update,Ovid MEDLINE(R) In-Process,Ovid MEDLINE(R) Publisher,CCTR,CDSR,Embase; records were retained] |
| 18 | 16 not 17 |
| 19 | from 13 keep 126-286 |
| 20 | from 13 keep 287-297 |
| 21 | 18 or 19 or 20 |
| 22 | remove duplicates from 21 |

SCOPUS

| 1 | TITLE-ABS-KEY ( ( ( exercis*  OR  training )  W/1  ( breathing  OR  respirat*  OR  ventilatory ) ) ) |
| --- | --- |
| 2 | TITLE-ABS-KEY ( "inspiratory muscle training" ) |
| 3 | 1 or 2 |
| 4 | TITLE-ABS-KEY ( ( ( coronary  OR  "aorta coronary"  OR  "aorto coronary"  OR  aortocoronary  OR  heart  OR  cardiac )  W/1  ( anastomosis  OR  bypass  OR  shunt*  OR  graft*  OR  surgery ) ) ) |
| 5 | 3 and 4 |
| 6 | INDEX(embase) OR INDEX(medline) OR PMID(0* OR 1* OR 2* OR 3* OR 4* OR 5* OR 6* OR 7* OR 8* OR 9*) |
| 7 | 5 not 6 |
| 8 | DOCTYPE(ed) OR DOCTYPE(bk) OR DOCTYPE(er) OR DOCTYPE(no) OR DOCTYPE(sh) OR DOCTYPE(ch) |
| 9 | 7 not 8 |
| 10 | LANGUAGE(english) |
| 11 | 9 and 10 |
